# Assessing Physician and Patient Agreement on Whether Patient Outcomes Captured in Clinical Progress Notes Reflect Treatment Success: Cross-Sectional Study

**DOI:** 10.2196/60263

**Published:** 2025-01-23

**Authors:** Sarah B Floyd, Jordyn C Sutton, Marvin Okon, Mary McCarthy, Liza Fisher, Benjamin Judkins, Zachary Cole Reynolds, Ann Blair Kennedy

**Affiliations:** 1 Department of Public Health Sciences Clemson University Clemson, SC United States; 2 Patient Engagement Studio University of South Carolina Greenville, SC United States; 3 Department of Orthopaedic Surgery Prisma Health Greenville, SC United States; 4 School of Medicine University of South Carolina Greenville, SC United States

**Keywords:** patient outcomes, proximal humerus fracture, patient involvement, orthopaedic medicine, clinical progress notes

## Abstract

**Background:**

It remains unclear if there is agreement between physicians and patients on the definition of treatment success following orthopedic treatment. Clinical progress notes are generated during each health care encounter and include information on current disease symptoms, rehabilitation progress, and treatment outcomes.

**Objective:**

This study aims to assess if physicians and patients agree on whether patient outcomes captured in clinical progress notes reflect a successful treatment outcome following orthopedic care.

**Methods:**

We performed a cross-sectional analysis of a subset of clinical notes for patients presenting to a Level-1 Trauma Center and Regional Health System for follow-up for an acute proximal humerus fracture (PHF). This study was part of a larger study of 1000 patients with PHF receiving initial treatment between 2019 and 2021. From the full dataset of 1000 physician-labeled notes, a stratified random sample of 25 notes from each outcome label group was identified for this study. A group of 2 patients then reviewed the sample of 100 clinical notes and labeled each note as reflecting treatment success or failure. Cohen κ statistics were used to assess the degree of agreement between physicians and patients on clinical note content.

**Results:**

The average age of the patients in the sample was 67 (SD 13) years and 82% of the notes came from female patients. Patients were primarily White (91%) and had Medicare insurance coverage (65%). The note sample came from fracture-related encounters ranging from the second to the tenth encounter after the index PHF visit. There were no significant differences in patient or visit characteristics across concordant and discordant notes labeled by physicians and patients. Among agreement levels ranging from poor to perfect agreement, physician and patient evaluators exhibited only a fair level of agreement in what they deemed as treatment success based on a Cohen κ of 0.32 (95% CI 0.10-0.55; *P*=.01). Furthermore, interpatient and interphysician agreement also demonstrated relatively low levels of agreement.

**Conclusions:**

The findings suggest that physicians and patients demonstrated low levels of agreement when assessing whether a patient’s clinical note reflected a successful outcome following treatment for a PHF. As low levels of agreement were also observed within physician and patient groups, it is clear the definition of success varied highly across both physicians and patients. Further research is needed to elucidate physician and patient perceptions of treatment success. As outcome measurement and demonstrating the value of orthopedic treatment remain important priorities, it is important to better define and reach a consensus on what treatment success means in orthopedic medicine.

## Introduction

In 1910, Ernest Amory Codman, an orthopedic surgeon, advocated for the concept of studying the “end result,” or the idea that every surgeon should follow patients long enough to evaluate whether the treatment they received was successful [1]. Early on, as surgeons began adopting Codman’s end result approach, physician-reported measurement of individual patient outcomes (eg, mortality, surgical complications, and degrees of range of motion) became the standard method to evaluate the success of orthopedic treatment. However, since that time, health care has continued to increase its appreciation of the patient’s perspective on outcome achievement, and patient preferences for outcomes following care [2-6]. As outcome measurement and demonstrating the value of orthopedic treatment are becoming an increasing priority [7,8], it is important to better elucidate what treatment success means in orthopedic medicine [9,10]. To date, it remains unclear if physicians and patients share the same definition of treatment success following orthopedic care.

The electronic health record (EHR) system is the primary tool to document and store records of patient encounters in hospitals and outpatient clinics in the United States [11-13]. Clinical progress notes are generated for each encounter that patients have with their physician or health care provider. These contain rich information on current disease symptoms, rehabilitation progress, and unexpected complications [14]. Unstructured progress notes produce a record of a patient’s history, physical findings, medical reasoning, and patient care and reveal distinct trajectories of patient outcomes after treatment [13,15,16]. In successful cases, the progress note documents the degree of improvement or relief experienced and reported by patients [17]. Conversely, when symptoms have not been resolved, are lingering, or when subsequent complications have arisen, these ongoing patient complaints and persistent treatment use are documented in the notes [18]. Clinical progress notes offer an opportunity to assess a range of outcome states and evaluate if physicians and patients have similar definitions of success following medical treatment for an orthopedic condition. Furthermore, the secondary use of EHR data is rapidly expanding, including the use of natural language processing and large language models to analyze unstructured clinical text [19-25]. One potential application of these methods includes using clinical notes as a data source to evaluate the success of orthopedic treatment. However, to correctly apply this method, a gold-standard definition of treatment success must be identified.

The objective of this paper was to assess agreement between patients and physicians on whether patient outcomes documented in clinical progress notes reflected successful or nonsuccessful treatment outcomes for patients receiving follow-up care for a leading shoulder condition, an acute proximal humerus fracture (PHF).

## Methods

### Study Sample

This was a cross-sectional analysis of a subset of progress notes from a larger study. The study included adult patients presenting in person to a Level-1 Trauma Center and Regional Health System for an acute PHF between January 1, 2019, and December 31, 2021. The index visit was defined as the first diagnosis at any health system site for PHF during the study period, with no previous visits for PHF within a year of the index visit. We identified all health system encounters (hospital encounters, office visits, etc) with a diagnosis of PHF or shoulder pain from the index PHF visit to 365 days after the index PHF visit. Of those encounters, we took the progress note from the last in-person office visit for PHF-related care, defined as a visit with a diagnosis of PHF (International Statistical Classification of Diseases and Related Health Problems 10th Revision [ICD10]: S42.2XXX) or shoulder pain (ICD10: M45.2XXX) to occur before 365 days postindex. This resulted in 1 note per person.

Patients were excluded from the study if they were less than 18 years of age, did not have at least 1 office visit with a diagnosis of PHF or shoulder pain that occurred 45 days or more days after the index visit, or if their last office visit was less than 500 characters. A minimum of 45 days after the index was used as this is the minimal time needed for healing of a PHF, before which treatment success cannot be assessed. The larger study included a sample of 1000 patients meeting these inclusion criteria. For this study, a sample of 100 progress notes was used to assess agreement between physicians and patients on their perceptions of treatment outcomes captured in the clinical notes. This study was approved by the Prisma Health Institutional Review Board (1924627-1).

### Outcome Label Development Process

The University of South Carolina Patient Engagement Studio (PES) brings together patients, caregivers, community groups, health system innovators, clinicians, and academic researchers to produce meaningful research that advances health outcomes. The PES membership includes over 100 patients with diverse backgrounds and clinical experiences from across the United States trained to provide feedback and collaborate with research teams [26-28]. PES staff members assembled a panel of 5 patients all of whom had a previous orthopedic experience including a joint injury of the shoulder, wrist, or ankle. These patients experienced a mix of surgical and nonsurgical management for their condition. Specific demographics of the panel are not shared per PES policy as these patients are consultants rather than study participants. PES staff members facilitated the senior author (SBF) to lead 3 sessions to codevelop a range of outcome states following orthopedic treatment. Together, the PES members and senior author defined 4 distinct outcome states that spanned the range of outcomes patients could experience following treatment for PHF.

**Figure 1:**
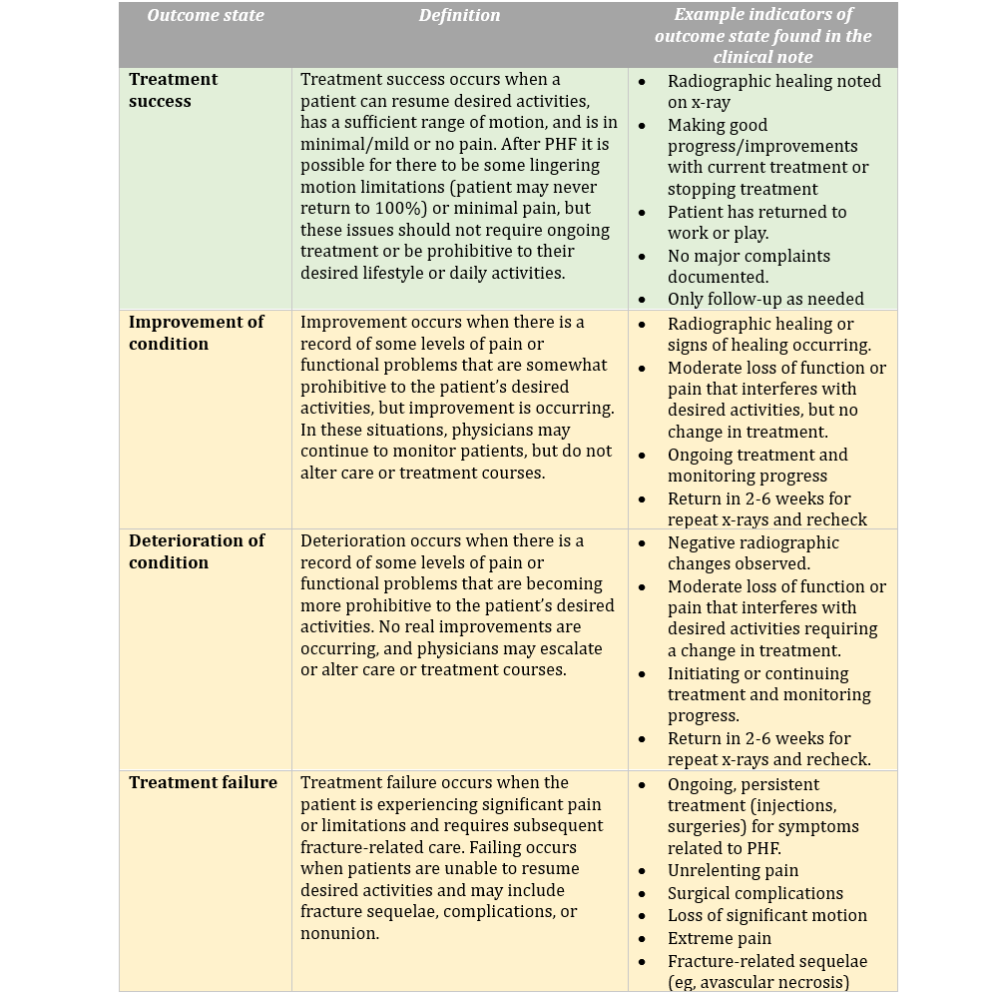
Treatment Outcome States, Definitions and Indicators Developed by Patient Engagement Studio and Research Team Members.

Figure 1 contains the 4 distinct outcome states, associated definitions, and indicators. The 4 outcome states included “Treatment Success” which is defined as patients resuming desired activities, achieving a sufficient range of motion, and no more than minimal or mild pain; “Improvement of Condition” included cases where there was a record of some level of pain or functional problems, but improvement of the condition was occurring; “Deterioration of Condition” occurred when there was a record of some level of pain or functional problems that were becoming more prohibitive to the patient’s desired activities and no improvement was occurring; and “Treatment Failure” occurred when the patient was experiencing significant pain or limitations and required subsequent fracture-related care for fracture sequelae, complications, or nonunion. These 4 outcome state labels were available to patients and physician evaluators when labeling each note.

### Note Labeling Process

#### Physician Evaluators

A total of 4 orthopedic residents were recruited to participate in the note-labeling process as part of the larger study. Each orthopedic resident received a 1-hour training on the study objective and outcome state labels. Residents were instructed to assess the current outcome state reflected in the note. The physician evaluators included 3 male and 1 female orthopedic residents, each of which had a minimum of 2 years of residency experience. When discordance occurred between residents’ labels, an attending orthopedic surgeon and the Chair of the Department of Orthopaedic Surgery served as the final note evaluator. REDCap (Research Electronic Data Capture; Vanderbilt University) [29,30] was used to organize and store physician labels for each note. From the full dataset of 1000 labeled notes, a stratified random sample of 25 notes from each outcome label group was identified, and the note sample (N=100) for patient labeling was created.

#### Patient Evaluators

We recruited 2 patients from the PES to participate in this study. Both patients were female and had personal orthopedic experience including upper and lower extremity conditions, but their personal clinical data were not included in our study sample. The patient evaluators brought both experiential expertise from their personal musculoskeletal conditions and specialized research training, enabling them to contribute effectively to this study. This aligns with current best practices in patient engagement, which emphasize the value of relevant patient perspectives and training over the necessity for identical clinical conditions [31-34]. Similar to the physician evaluators, patient evaluators also received a 1-hour training on the study objective and outcome state labels. The training included a group review of example charts and common language used in medical charts. In addition, we trained patients in the subjective, objective, assessment, and plan sections [14] format typically used in medical documentation to increase their familiarity with navigating a medical chart. All clinical progress notes were redacted to conceal patient identifiers before patient review.

Both patient evaluators reviewed all 100 notes and provided labels. In addition to the 4 outcome state labels, a label of “Insufficient” was available for patient evaluators for notes deemed to have insufficient information to assign an outcome label. When discordance occurred between patient evaluators, the Program Manager of the PES (KP) served as the final note evaluator. After review by the Program Manager, all notes had a final label, and all labels of “insufficient” were resolved.

### Patient and Visit Characteristics

Patient characteristics associated with the 100 clinical notes included in the analysis were extracted from the health system EHR, Epic, and included patient age, sex, race, and insurance provider. Patient characteristics were identified from the index PHF visit. In addition, visit characteristics, including days between the index visit and visit date for the clinical note, the number of PHF-related encounters, surgical treatment use, and note length, were also included in the analysis. Patients receiving surgery were defined as those patients undergoing reverse shoulder arthroplasty, hemiarthroplasty, or open reduction internal fixation between the index and 365 days.

### Statistical Analysis

The 4 outcome labels were aggregated into a binary classifier representing treatment success or failure. Success was represented by notes labeled “Treatment Success.” The 3 remaining labels, including “Improvement of condition,” “Deterioration of condition,” and “Treatment Failure” were grouped into the Treatment failure group. Treatment failure was comprised of all labels with documentation of lingering, symptomatic problems requiring ongoing care.

Agreement between physicians and patients was calculated across binary groups of treatment success or failure. Discordant labels were defined as notes with differing outcome states provided by the respective labelers. Cohen κ statistics were used to assess the degree of agreement between patient evaluators, as well as the degree of agreement between physician and patient labels. In addition, physician agreement was reported for the larger sample of 1000 notes and was assessed using Fleiss κ [35]. We used the benchmarks for agreement for categorical data as described by Landis and Koch [36], where 0.00-0.20, 0.21-0.40, 0.41-0.60, 0.61-0.80, and 0.81-1.00 indicate poor, fair, moderate, substantial, and almost perfect agreement, respectively. A Bangdiwala agreement chart is presented to display the agreement between physician and patient labels [37].

Descriptive analyses were used to assess the characteristics of the progress note sample. Mean and SD were reported for parametric variables. Median and IQR (25% and 75%) were reported for nonparametric variables. Two-sample *t* test, Wilcoxon-Mann-Whitney, and chi-square tests were used to assess differences in concordant and discordant notes. Analyses were performed with SAS (version 15.2; SAS Institute), R studio (R Core Team), and Microsoft Excel.

## Results

### Progress Note Characteristics

The sample of 100 progress notes for this study came from patients treated across 24 departments and 54 distinct physicians within one regional health system. The 24 departments from which the notes were identified included 21 orthopedic practices or departments, 2 family medicine, and 1 pain management clinic. Notes were authored by both physicians and advanced practice providers. Of the 41 physicians, 35 (85%) specialized in orthopedics, whereas the remaining 6 (15%) were specialists in family medicine. In addition to the 41 physicians, 13 advanced practice providers completed notes and 10 (77%) of these providers specialized in orthopedics, while the remainder had other specialty training including general surgery and pain medicine.

The average age of the patient was 67 (SD 13) years and 82% of the notes came from female patients. Patients were primarily White (91%) and had Medicare insurance coverage (65%). The note sample came from fracture-related encounters ranging from the second to the 10th encounter after the index PHF visit, with a median time of 115 (IQR 73-215) days after the index. The progress notes text lengths ranged from 981 to 15,297 characters with a median length of 5098 (IQR 2846-7810) characters. There was no significant difference in progress note characteristics across concordant and discordant notes (Table 1).

**Table 1:** Patient and visit characteristics of the clinical progress note sample presented by patient and physician agreement (N=100). Mean and SD were reported for parametric variables. Median and IQR (25% and 75%) are reported for nonparametric variables. A 2-sample *t* test was used for parametric variables and the Wilcoxon-Mann-Whitney test was used for nonparametric comparisons.

Patient characteristics		Total sample (N=100)	Concordant notes (n=78)	Discordant notes (n=22)	*P* value	
Patient age (years), mean (SD)		67 (13)	67 (13)	68 (13)	.73	
**Patient sex, n (%)**						.22
	Male	18 (18)	16 (20)	2 (9)	—^a^	
	Female	82 (82)	62 (79)	20 (90)	—	
**Patient race, n (%)**						.72
	White	91 (91)	71 (91)	20 (91)	—	
	Black	5 (5)	3 (4)	2 (9)	—	
	American Indian or Alaskan	1 (1)	1 (1)	0 (0)	—	
	Hispanic	1 (1)	1 (1)	0 (0)	—	
	Unknown	2 (2)	2 (3)	0 (0)	—	
**Insurance provider, n (%)**						
	Medicare	65 (65)	51 (65)	14 (64)	.44	
	Medicaid	7 (75)	7 (9)	0 (0)	—	
	Private	21 (21)	15 (19)	6 (27)	—	
	Other	7 (7)	5 (6)	2 (9)	—	
**Visit characteristics**						
	Days from index, median (IQR)	115 (73-215)	113 (74-219)	115 (65-170)	.65	
	PHF^b^-related encounter, median (IQR)	4 (3-6)	4 (3-6)	4 (3-6)	.44	
	Patient treated surgically, n (%)	25 (25)	21 (27)	4 (18)	.40	
	Note character length, median (IQR)	5098 (2846-7810)	5202 (2901-8155)	4320 (2672-6428)	.19	

^a^Not applicable.

^b^PHF: proximal humerus fracture.

### Agreement Between Patients

Both patient evaluators were assigned the full sample of 100 notes to review and label. Of the 100 notes, 34 notes were discordant between patient evaluators. A total of 23 of the discordant labels were between success and failure labels between patient evaluators. In addition, there were a total of 11 cases (across patient evaluators 1 and 2) that received a label of “insufficient.” There was a statistically significant level of agreement between the 2 patient evaluators (Cohen κ=0.41, 95% CI 0.23-0.59; *P*<.001), and the strength of agreement was classified as moderate, according to Landis and Koch. Tables 2 and 3 show the agreement in note labels between patient evaluators and physicians and patient evaluators.

**Table 2:** Agreement in note labels between patients (N=100).

Patient rater 1	Patient rater 2					Agreement
	Success	Failure	Indeterminate^a^	Total		
Success	15	3	1	19	Moderate (κ=0.41)^b^	
Failure	20	51	8	79		
Indeterminate^a^	0	2	0	2		
Total	35	56	9	100		

^a^A label of indeterminant was available for use by patient evaluators for notes deemed to have insufficient information for a label. Notes labeled as insufficient were reviewed by the PES Manager for final label assignment. After final review, all notes had a final label, and all labels of insufficient were resolved before future analysis.

^b^Cohen κ used to assess agreement. 0.00-0.20, 0.21-0.40, 0.41-0.60, 0.61-0.80, and 0.81-1.00 indicate poor, fair, moderate, substantial, and almost perfect agreement.

**Table 3:** Agreement in note labels between physicians and patients (N=100).

Physician labels	Patient labels			Agreement
	Success	Failure	Total	
Success	11	14	25	Fair (κ=0.32)^a^
Failure	8	67	75	
Total	19	81	100	

^a^Cohen κ used to assess agreement. 0.00-0.20, 0.21-0.40, 0.41-0.60, 0.61-0.80, and 0.81-1.00 indicate poor, fair, moderate, substantial, and almost perfect agreement.

### Agreement Between Physicians and Patients

**Figure 2:**
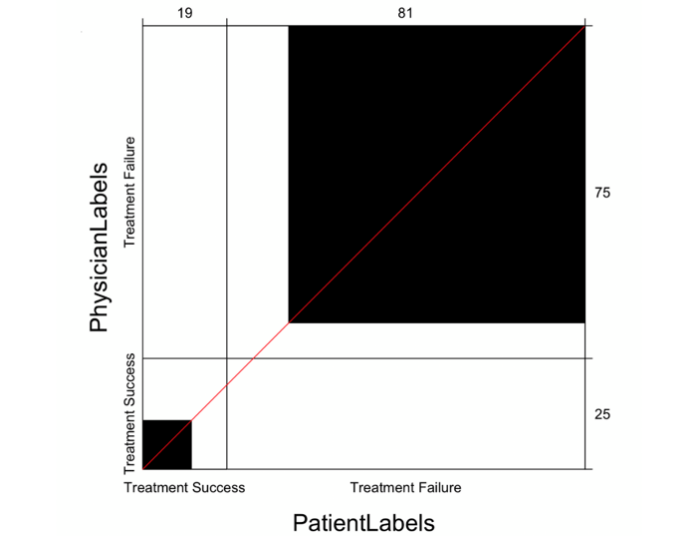
Bangdiwala agreement chart for physician and patient note labels (N=100). Bangdiwala chart used to assess agreement between patients and physician’s indications of treatment success or treatment failure from analyzed clinical notes. Black boxes indicate overlap of agreement.

A total of 22 notes were discordant between physicians and patient evaluators. Of the 25 notes labeled as treatment success by orthopedic surgeons, 11 notes were also labeled as treatment success by patients. The remaining 14 treatment success notes were labeled as treatment failure by patient evaluators. Of the 75 notes deemed as treatment failure, 67 were also labeled as treatment failure by patient evaluators. There was a statistically significant level of agreement between orthopedic physicians and patient evaluators (Cohen κ=0.32, 95% CI 0.10-0.55; *P*=.01). The strength of agreement between patients and physicians was classified as fair, according to Landis and Koch. Figure 2 includes a Bangdiwala chart used to display agreement between patients’ and physicians’ assessment of treatment success or treatment failure from analyzed clinical notes.

Although not the focus of this paper, physician agreement was assessed using the larger sample of 1000 notes. Agreement between physicians was assessed using Fleiss κ and agreement between orthopedic physicians was moderate (Fleiss =0.49, 95% CI 0.30-0.68; *P*=.04).

## Discussion

### Principal Findings

The objective of this paper was to assess if physicians and patients agree in their assessment of whether patient outcomes in clinical progress notes reflected a successful treatment outcome following orthopedic care. This is an important question to answer for the field of orthopedic medicine which has experienced a paradigm shift in the way in which outcomes are assessed [3,38,39]. Outcome assessment in orthopedics dates back over 100 years. Early on, physician-reported measurement of individual patient outcomes was the standard method by which to evaluate the outcomes of orthopedic care. However, today outcome measurement directly from a patient’s perspective is viewed as the gold standard in orthopedic medicine [39,40]. We were interested in exploring if patients and physicians have similar definitions of what successful outcomes mean following orthopedic treatment.

In our analysis, we had patients and physicians review a subset of 100 clinical progress notes and label the note as a successful or unsuccessful outcome. We found that physicians and patients only exhibited a fair level of agreement in what they deemed as treatment success documented in progress notes. In addition, we found that physicians and patients had higher levels of agreement in what represented treatment failure compared with treatment success. Furthermore, interpatient and interphysician agreements also demonstrated relatively low levels of agreement, signaling that even within patients and physician groups, the definition of success is not clearly defined or agreed upon.

### Comparison to Previous Work

A potential explanation for the low level of agreement between patients and physicians may simply be that patients and physicians have different expectations following care. Our findings might signal that physicians have different expectations of patient’s capabilities following a serious upper extremity injury, such as PHF [41,42]. For other orthopedic treatments, it has been reported that patient expectations may be greater than a physician’s expectations [43]. For example, in total hip and knee arthroplasty, most patients had higher expectations for recovery than their surgeon [43]. This might explain why over half of the notes labeled as treatment success by orthopedic surgeons were labeled as treatment failure by patients. Patients appeared to have a more stringent definition of success compared with physicians. Although not the goal of our study, this finding does emphasize the importance of shared decision-making within orthopedic encounters, to ensure patients have realistic expectations of outcomes following care [44].

An alternative explanation for our finding could be that physicians and patients define success differently. In a study assessing patient-physician agreement on the management of musculoskeletal injuries and pain associated with those injuries, authors found that patients and physicians prioritize different goals when assessing a patient’s treatment outcome [4,45]. For example, physicians may have a more clinically based definition of treatment success driven by objective measures such as radiographic measures of healing and degrees of range of motion, whereas patients may be more focused on the ways in which outcomes like pain and joint function relate to daily capabilities and quality of life [5].

We found that physicians and patients had higher levels of agreement in what represented treatment failure compared with treatment success. Other studies measuring patient and physician agreement following orthopedic surgery concluded that patients and physicians agreed more when the patient had good health outcomes [4,46,47]. These conclusions are not consistent with our study findings. We found that physicians and patients were in agreement for a larger share of the treatment failure notes, compared with the treatment success notes. It is our belief that treatment failure is more clear-cut (eg, surgical complications, persistent pain, and fracture nonunion), whereas treatment success is more variable and patient-specific. Consequently, it may be easier to recognize when outcomes are unfavorable, but pinpointing a positive outcome proves challenging due to the variability and outcome preferences across individual patients [48,49]. Furthermore, we believe the concept of a patient-specific definition of success is supported by the moderate level of agreement we observed between patients. This signals that even among patients, there is a differential evaluation of an acceptable outcome. There is not 1 singular definition of treatment success, instead, treatment success depends on an individual patient’s lifestyle and desired goals. Finally, even among physicians, we still observed relatively low levels of agreement, signaling that the definition of success remains unclear across physicians.

### Limitations

Our work has several limitations that should be acknowledged. First, we used a relatively small sample of progress notes from 1 clinical condition that lacks patient diversity. Furthermore, our results are highly reflective of the small sample of physicians and patient evaluators who completed the labeling. Next, we were unable to assess the characteristics of treating physicians who authored the progress notes. It is possible physician characteristics like subspecialty training, years of experience, and so on. may explain some of the discordance in note labels. In addition, we worked with resident physicians who may be less experienced in assessing patient outcomes following care. This could affect physician agreement, as well as physician-patient agreement results. Also, the way in which we aggregated patient labels may influence the level of agreement we observed. For example, more categories could potentially lead to lower concordance among evaluators. Finally, it is possible that as nonmedically trained individuals patient evaluators’ labeling may have been influenced by their lack of medical training.

### Future Directions

Although outside the scope of this work, there remain questions surrounding the accuracy of clinical notes. There are mixed reports of the accuracy, completeness, and quality of progress note content [50-53]. Multiple studies have found that health care professionals produce accurate documentation for concrete and overt symptoms, such as range of motion and impaired physical functioning [54]. However, it must be acknowledged that we did not directly assess the accuracy of physician reporting of patient outcomes captured in the clinical notes. Secondary use of EHR data is rapidly expanding, including the use of natural language processing and large language models to analyze unstructured clinical text [19-25]. One potential use could be to use clinical notes to evaluate the success of orthopedic treatment. However, to appropriately assess and classify outcomes as either successful or unsuccessful, the accuracy of clinical notes must be assessed.

In addition, as we work to continue to understand the concept of treatment success in orthopedic medicine, it may be helpful to conduct follow-up interviews with physicians and patients as they conclude the labeling process. This could reveal a deeper understanding of each perspective on what treatment success means. Furthermore, we anticipate that future work will incorporate multiple clinical notes across the episode of care to capture a more complete outcome assessment, as interim visits may reveal incremental improvements before the final visit.

### Conclusion

The objective of this study was to assess if physicians and patients agree on whether patient experiences captured in clinical progress notes reflect a successful patient outcome following orthopedic treatment. In performing a cross-sectional analysis of clinical progress notes from an acute follow-up of patients treated for a PHF, we found fair agreement between patients’ and physicians’ assessments of patient outcomes reflecting treatment success. These results indicate that patients and physicians do not fully agree on what constitutes treatment success. Our findings emphasize the need to analyze both patient and physician perspectives when determining treatment success. Further research is needed to examine how different perceptions of treatment success may influence outcome development and use in orthopedic medicine.

## Data Availability

The datasets generated during and/or analyzed during this study are available from the corresponding author on reasonable request.

## Abbreviations

EHR: electronic health record
ICD10: International Classification of Diseases, 10th Revision
PES: Patient Engagement Studio
PHF: proximal humerus fracture
REDCap: Research Electronic Data Capture
